# Imaging Lymphoma With F-18 Fluorodeoxyglucose PET-CT: What Should Be Known About Normal Variants, Pitfalls, and Artifacts?

**DOI:** 10.3389/fnume.2021.826046

**Published:** 2022-02-01

**Authors:** Mboyo Di Tamba Vangu, Jaleelat I. Momodu

**Affiliations:** Department of Nuclear Medicine and Molecular Imaging, Charlotte Maxeke Johannesburg Academic Hospital, University of the Witwatersrand, Johannesburg, South Africa

**Keywords:** FDG, Hodgkin, lymphoma, non-Hodgkin, PET/CT, pitfalls, variants

## Abstract

^18^F fluorodeoxyglucose ([F-18] FDG) PET-CT has gained popularity in the management of many types of malignancies. Today, imaging patients with lymphoma using of [F-18] FDG PET-CT not only is considered as a state-of-the-art tool but also has taken a central place for therapeutic decisions. In fact, accurate staging at diagnosis is imperative to prevent under treatment of individuals with advanced disease. In Hodgkin's lymphoma, in particular, the current role of interim [F-18] FDG PET imaging goes beyond speculations in the adaptation of different therapeutic strategies. Therefore, the use of such a critical imaging modality should go hand in hand with sound interpretation that provides accurate results. As the number patients referred for PET-CT continues to increase, imaging specialists should remain aware of the inherent limitations linked to the integrated imaging system that may introduce potential pitfalls related to the machine or the administered [F-18] FDG. Knowledge of the normal physiologic biodistribution of [F-18] FDG, its physiologic variants, and of all the potential pitfalls and artifacts is paramount to avoid misinterpretation. Recognition of the limitations of [F-18] FDG PET-CT will increase the confidence of practicing clinicians on the modality and impact positively on the management of patients. In this article, we will review the normal physiological variants, technical artifacts, and diagnostic pitfalls in lymphoma. Highlighting the limitations of [F-18] FDG PET-CT imaging should warn interpreting specialists to find measures that mitigate them and improve reporting results.

## Introduction

The popularity gained by positron emission tomography-computed tomography (PET-CT) with the use of ^18^F-fluorodeoxyglucose ([F-18] FDG) in oncology for the management of malignancies is not to demonstrate anymore. In individuals affected with lymphoma, the modality is today at the center of both staging and therapy decision. PET-CT is currently the imaging standard technique in staging patients with Hodgkin's lymphoma (HD) and those with non-Hodgkin's lymphoma (NHL) (1). The Lugano classification and clinical guides recommend using [F-18] FDG PET-CT for routine staging of all FDG-avid nodal lymphomas for interim staging and at the end of chemotherapy to evaluate response (1–3). [F-18] FDG PET-CT imaging is also recommended as a baseline staging modality by the National Comprehensive Cancer Network (NCCN) guidelines for HD and diffuse large B-cell (DLBC) lymphoma as well as in many other forms of NHL (4, 5). The accuracy of PET-CT is great with higher sensitivity and specificity than conventional anatomical imaging for nodal and extranodal disease (1, 2). However, it must be noted that the sensitivity of PET-CT in lymphoma is much higher in the chest than that in the abdominal and pelvic regions (6). Because of its similar sensitivity and positive predictive value (PPV) to bone marrow biopsy (BMB) but at the same time having better sensitivity and negative predictive value (NPV), PET-CT may be used in place of BMB in HD and DLBC lymphoma (7). Since [F-18] FDG is the principal radiopharmaceutical used to image lymphoma with the use of PET-CT, this paper will provide a brief description on normal distribution and potential artifacts to avoid a lengthy repetition of information from other articles published in the same edition of the journal.

## Normal Distribution

[F-18] FDG is used in oncology imaging as an analog of glucose, and most malignant tumors are known to have increased glycolysis. Normal tissues and sites of inflammation also utilize glucose, thus making FDG a non-specific tracer of malignant tissue. Although the mechanism of uptake of [F-18] FDG is beyond the aim of this paper, it should be remembered that hyperglycemia may interfere with tumor uptake due to competition with glucose (8). Routinely, patients are asked to fast for at least 4–6 h prior to [F-18] FDG administration to minimize the impact of insulin levels and therefore improve tumor uptake (9). Since most patients fast for 4–6 h, it is sensible to advise them to take clear water before administration of FDG. However, glucose levels are routinely checked, and it is acceptable to inject FDG if levels are 200 mg/dl or less. Generally, imaging is performed at about 60 min after tracer administration to allow enough tumor uptake. Essentially, FDG uptake is seen throughout the body with variable degrees in different organs. The brain exhibits high uptake, the myocardium may still show high uptake despite fasting of 4–6 h, and urinary activity of excreted FDG is seen as high activity in kidneys, ureters, and bladder. Hence, patients are requested to empty their bladder before starting acquisition of images.

## Variants, Artifacts, and Pitfalls

It is important to recognize physiological variations of FDG uptake, technical artifacts, and potential pitfalls that may impact the interpretation of PET-CT in lymphoma. Because the usual imaging acquisition is similar for most malignant diseases and often includes the head down to mid-thigh region, physiological variants and technical artifacts are also similar. Variations on physiological uptake of FDG are well recognized (1, 10, 11), and some may mimic pathological findings.

**Figure 1:**
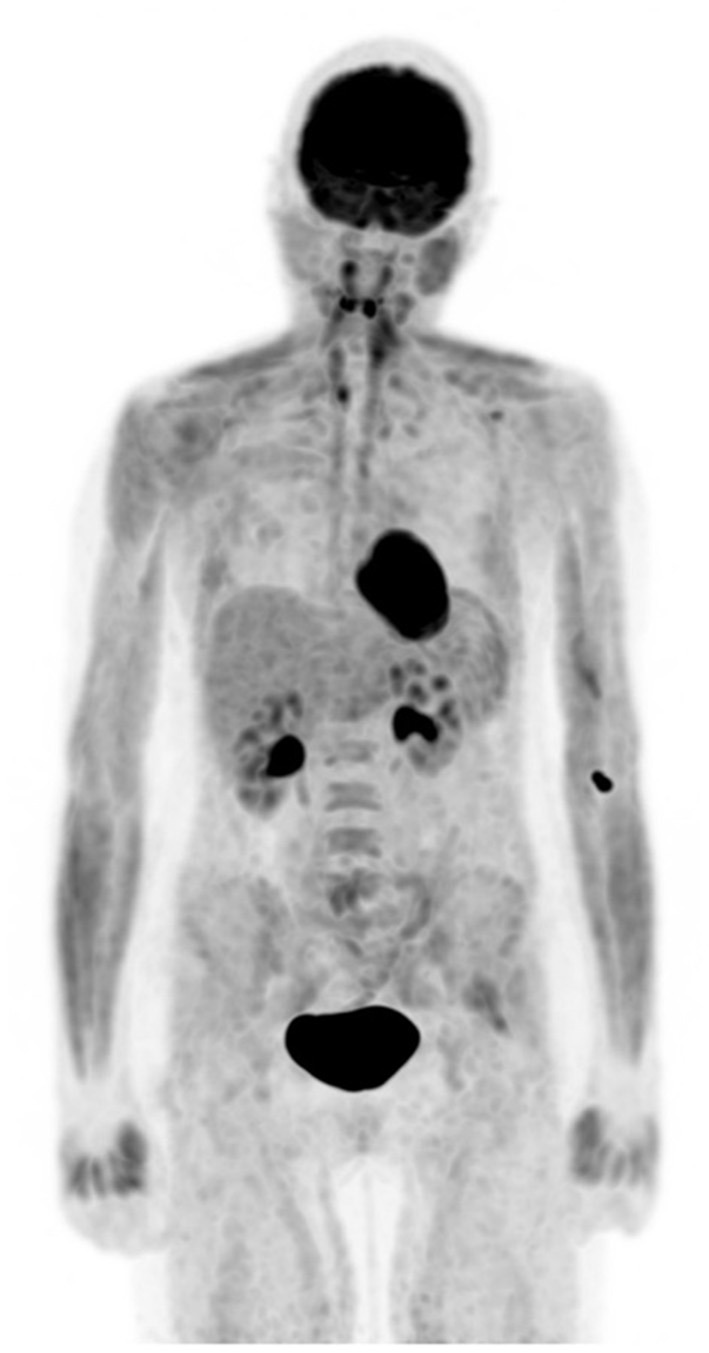
Altered biodistribution. FDG PET maximum intensity projection (MIP) image demonstrates diffuse increased muscular uptake in a patient who admits having a meal about an hour before coming for injection despite the knowledge of preparation for study.

Uptake in skeletal muscle is one of the most common alterations that should be recognized and is mainly due to either exercise or unwanted muscle activation from stress that increases FDG accumulation and impacts interpretation, as it may mimic pathology (Figure 1). Patients should therefore be advised to avoid exercise both before administration of [F18] FDG and in period following injection to prevent muscle uptake. Situations that will have similarly increased muscle uptake include non-fasting individuals, diabetic patients on insulin, and patients on corticosteroid treatment (1, 11, 12).

**Figure 2:**
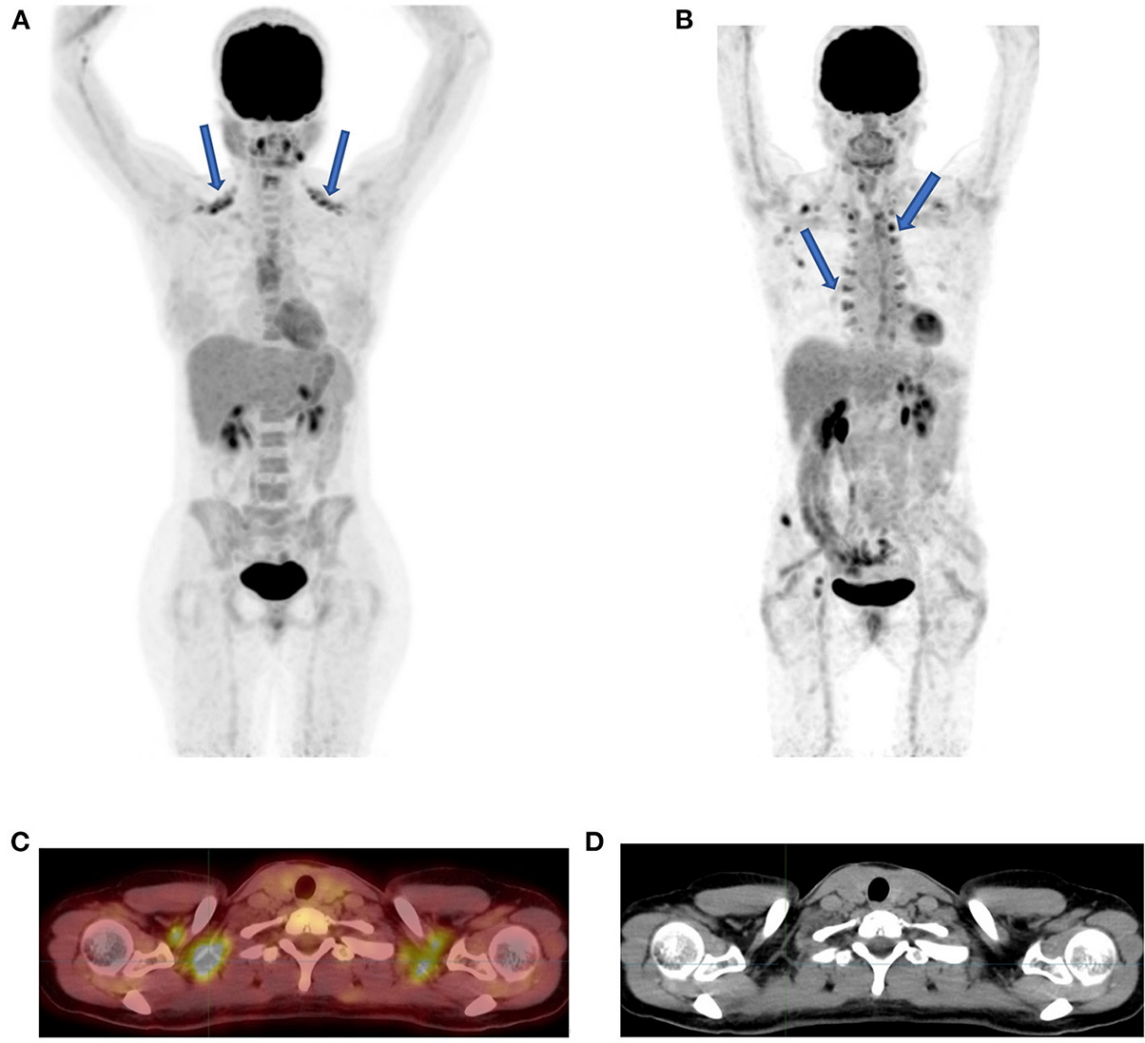
Brown fat uptake. FDG PET MIP image showing bilateral uptake in supraclavicular **(A)** and paraspinal fat **(B)** (solid arrows). The axial fused PET-CT **(C)** and CT **(D)** demonstrate that the FDG uptake has no CT corresponding tissue thus indicative of brown fat in a patient in **(A)** who was scanned during southern winter.

In patients with lower body mass and during winter seasons or cold days, patterns of symmetrical FDG uptake are common in the neck, supraclavicular, and paraspinal regions (Figure 2). The culprit for these patterns of FDG uptake has been identified as brown fat, a known organ of thermogenesis (11). Of the two forms of fat in the human body, white and brown, the latter generates heat when a human body is exposed to cold (1).

While the abovementioned three anatomical regions seem to be the prominent areas of visualized uptake in brown fat, attention should be paid to other areas of potential brown fat uptake that include the axilla, intercostal spaces, paracolic and parahepatic spaces, periphrenic space, and around the large vessels in the mediastinum (1). One should remember that decreasing adrenergic stimulation may minimize FDG accumulation in brown fat. Pharmacological intervention with oral diazepam or administration of propranolol and limiting exposure of patients to cold temperature are the proposed measures to decrease brown fat uptake (1, 11).

**Figure 3:**
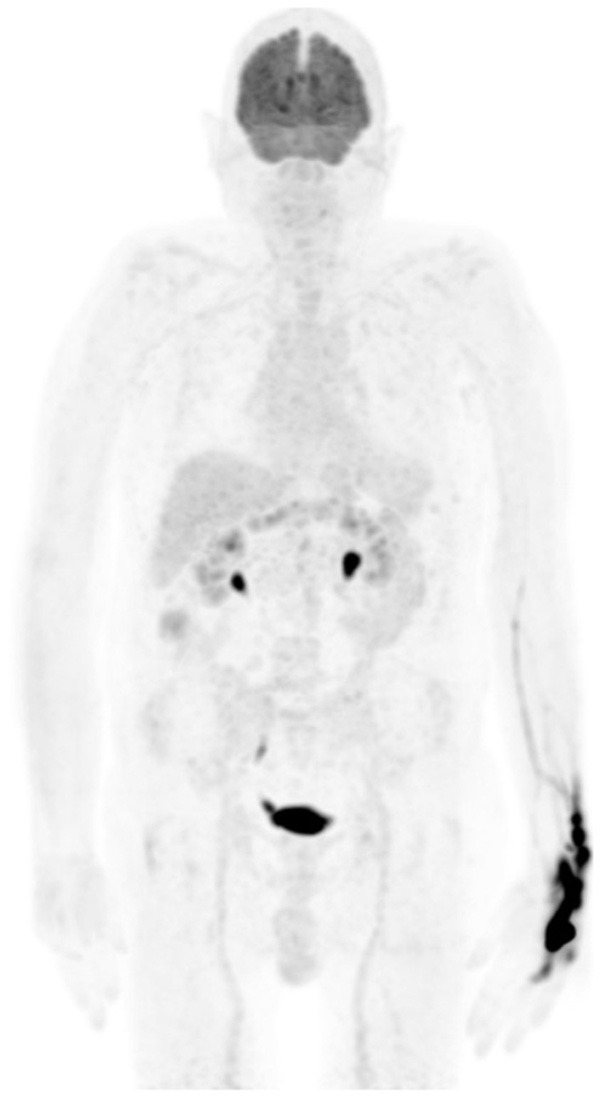
Extravasation uptake. MIP image from FDG PET shows a large area of intense uptake in the left wrist and hand due to injection in soft tissue. The imaging may be of adequate quality, but the calculated SUV of visualized pathology may be erroneous, and the interpreting physician must be aware of such for comparison with follow-up studies.

Knowledge of common artifacts may assist the interpreting physicians to decrease the number of studies that may require to be repeated and in the understanding of altered FDG distribution. Extravasation of FDG at the injection site must be recognized because a small amount of such activity may mimic a subcutaneous focal pathology or be drained in the adjacent lymph node station. However, a large amount of FDG extravasation in soft tissue may cause a significant artifact (Figure 3), and such great extravasation may interfere with the accuracy of calculated standard uptake values (SUVs) of the pathological areas of interest, as they may not reflect the true dosage of injected activity in the blood (11).

**Figure 4:**
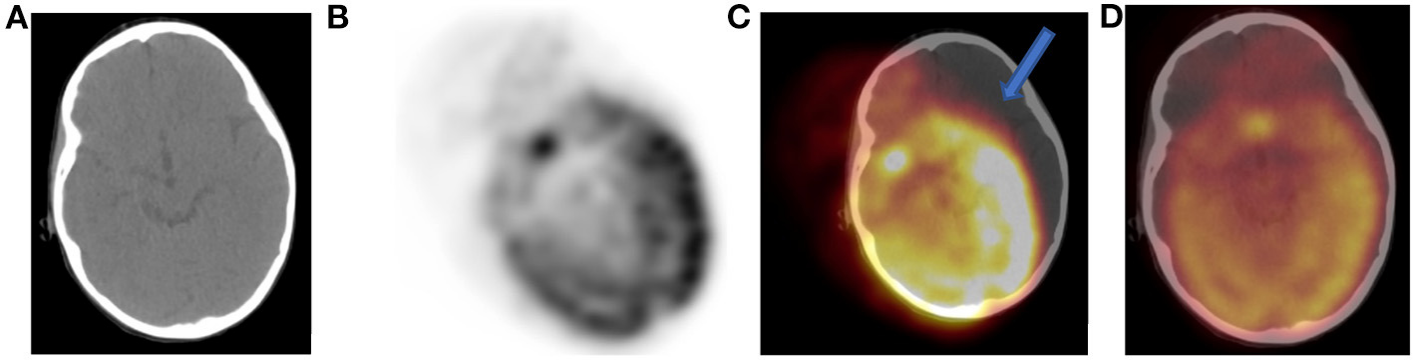
Tilt of CT **(A)** with gross misregistration artifact. Axial FDG PET **(B)** showing oblique position of brain uptake and fused PET-CT **(C)** images demonstrate brain FDG uptake erroneously localizing to the left outside skull (solid arrow). We used a correction software that is part of the package of our new digital PET-CT to reposition brain tissue within skull **(D)**. The artifact results from patient motion and changing position between CT and PET acquisition.

Motion artifacts may also occur due to patients changing position during PET acquisition. Because most current CTs part of integrated systems are fast, patients' motion during PET acquisition will result in misregistration (1, 11–14). Although respiratory gating in modern PET-CT scanners will minimize misregistration, attention must be paid near the diaphragm where significant motion will cause the FDG to project over anatomical areas that may mimic pathology. Correction of any misregistration is possible if recognized and with the use of current software packages that allow adjustment of date and correct alignment error (Figure 4).

**Figure 5:**
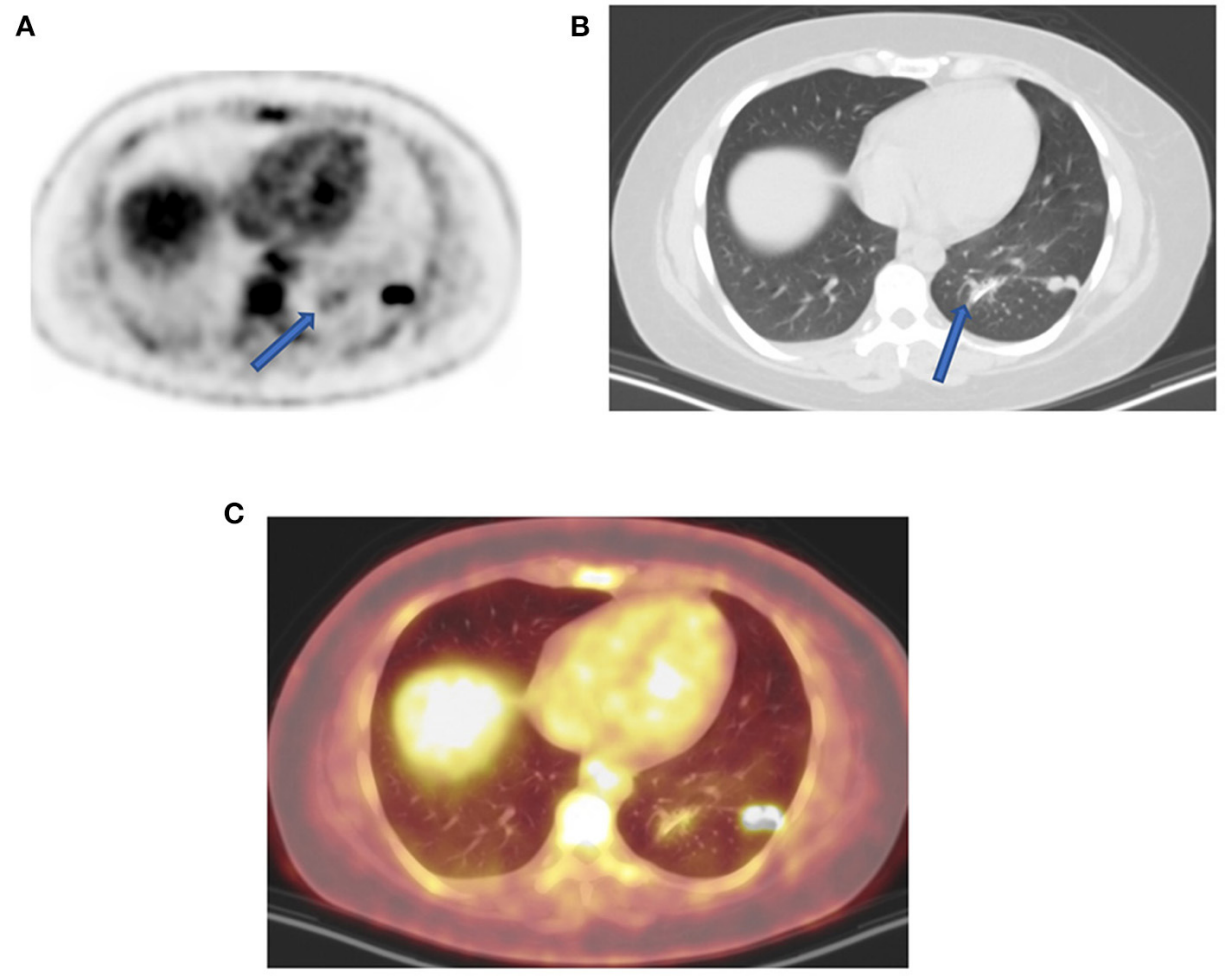
Uptake due to unusual attenuation correction. FDG PET axial **(A)** attenuation-corrected image shows intense FDG uptake in the posterior lateral aspect of the left lung and moderate uptake medially (arrow) caused by an artifact induced by attenuation correction. Corresponding lung window CT **(B)** shows sutures (arrow) from previous surgery (13 months earlier) as confirmed on combined PET-CT **(C)** corresponding plane. Without CT anatomy, the uptake caused by attenuation correction could mimic pulmonary pathology adjacent to real lung active nodules. In the presence of potential attenuating elements such metal implant, surgical sutures, and staples with unexplained FDG uptake, it is upon interpreting physicians to evaluate corresponding non-attenuation-corrected images to minimize reporting errors.

Movements from patients may also cause ring artifacts that result from misregistration of transmission (CT) and emission (PET) scanning. Attenuation-corrected artifacts are not less common and must be recognized. Metal implants and objects carried by patients and oral or intravenous contrast may create areas of false increased FDG uptake due to attenuation correction artifacts (Figure 5). It is thus advised to review the data set of non-attenuated images, as the artifacts are usually readily recognized as the false increased uptake disappears (1, 13, 14).

**Figure 6:**
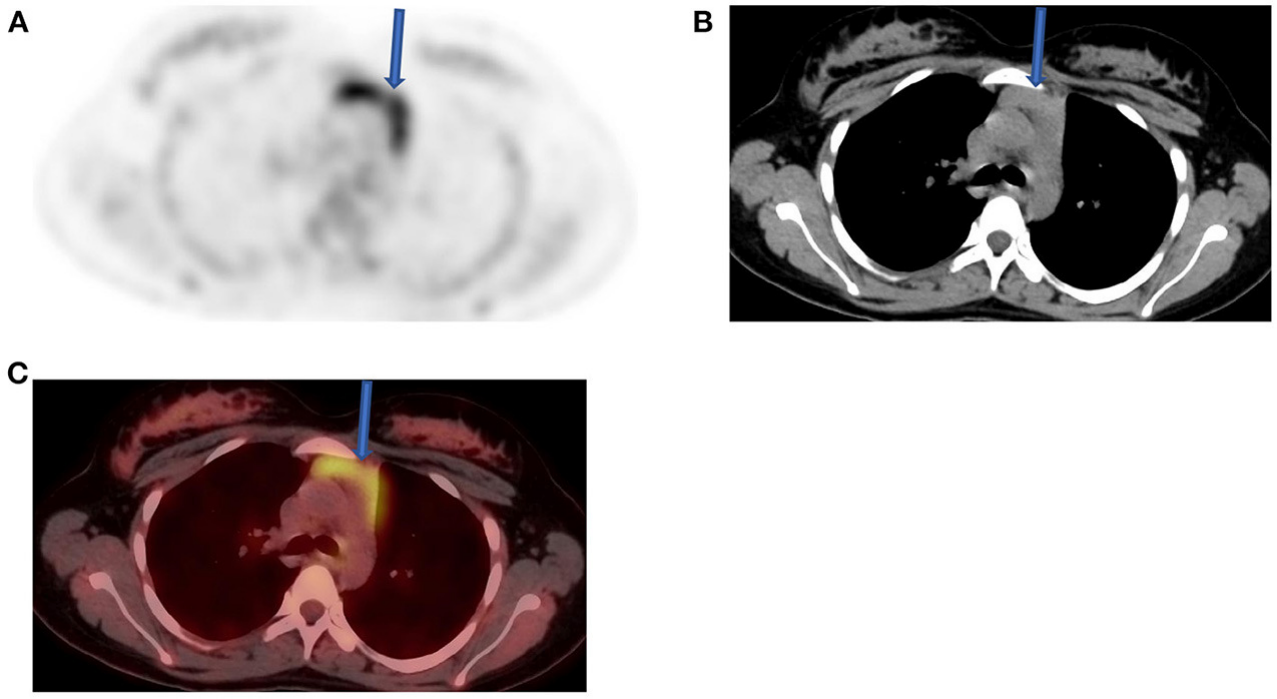
Thymus activity from a 12-year-old female who was diagnosed with Hodgkin's lymphoma (HD) from cervical lymph nodes. She was referred for a staging, and FDG/PET image **(A)** shows normal uptake in the thymus (solid arrow) with the expected inverted “V” shape that is consistent with a variant of normal. Both CT **(B)** and fused PET-CT **(C)** confirmed the nature of visualized change in the anterior mediastinum.

**Figure 7:**
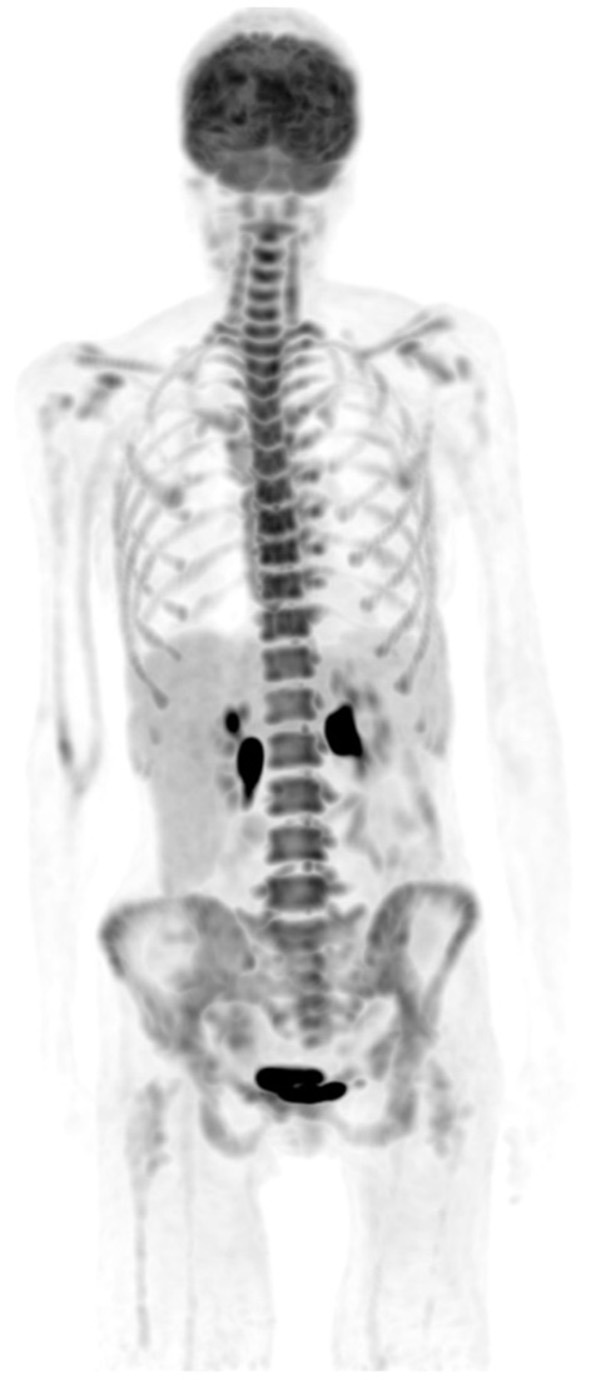
A 50-year-old female diagnosed with Hodgkin's lymphoma (HD) and received 3 cycles of R-CHOP. She was referred for interim evaluation of therapy. FDG PET MIP image shows evidence of marrow stimulation with diffuse increased uptake throughout the red marrow due to response to treatment. Patterns of diffuse FDG at interim or immediately following completion of therapy should not be interpreted as bone marrow involvement.

**Figure 8:**
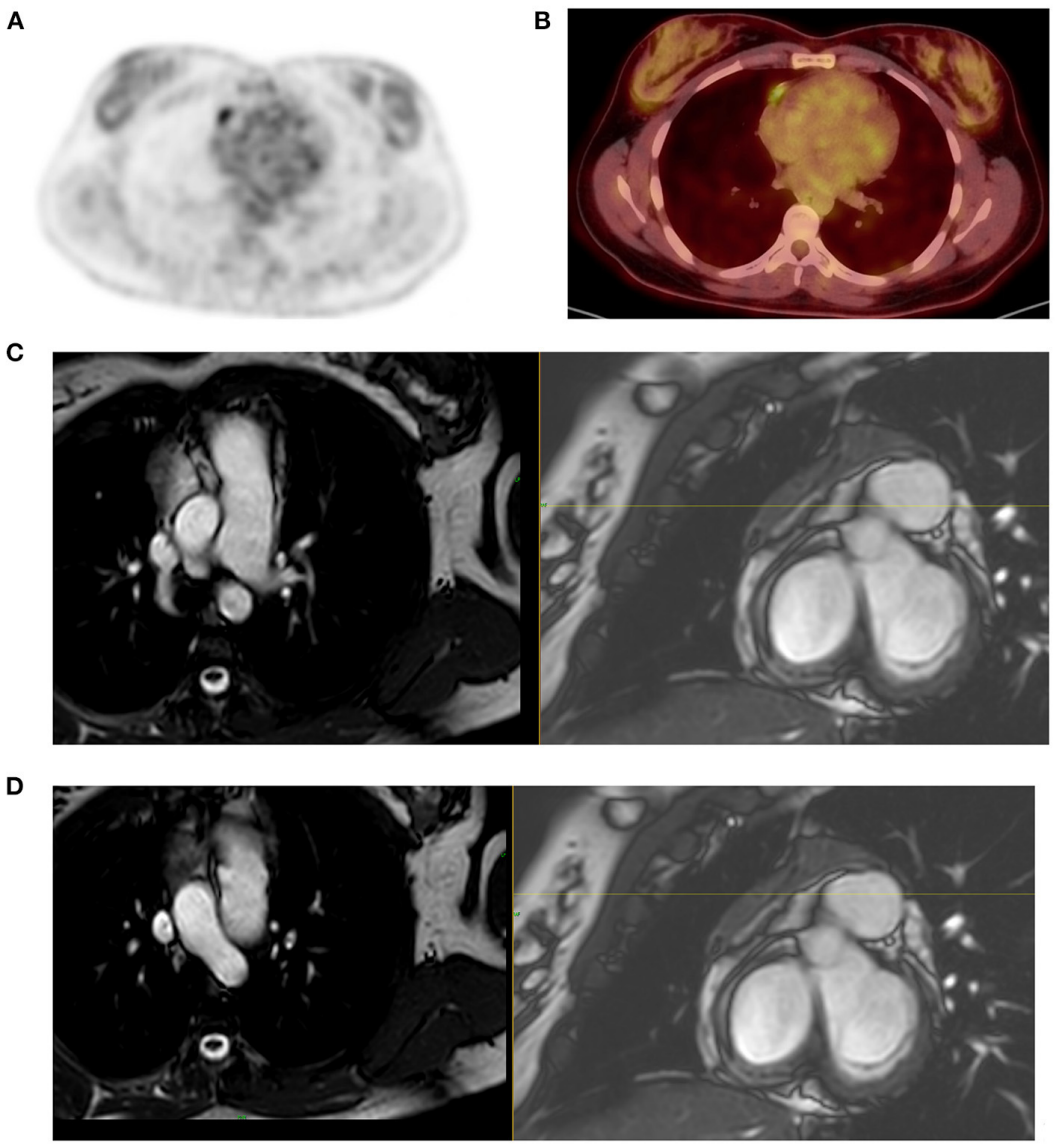
Mimic of active lymph node in post therapy PET-CT. A 22-year-old female patient treated for Hodgkin's lymphoma (HD) and had PET-CT scan for therapy evaluation outside our academic platform. The study was reported as showing a new FDG active lymph node in the mediastinum, lateral to the right heart thus consistent with progressive disease per Lugano criteria. She received more chemotherapy and referred to us for further evaluation 6 months after previous evaluation. FDG PET MIP image demonstrated focal mediastinal uptake **(A)** that was lateral to the right heart **(B)** that was described in the study done 6 months earlier. Noting the persistent activity after further treatment and the linear pattern of the uptake **(B)**, we suspected possible uptake in atrial appendage and requested correlation with cardiac MR to exclude active mediastinal lymph node as the cause of uptake and its implication on the management of this young patient. MRI showed **(C,D)** an enhancing triangular anterior mediastinal tissue extends along the right heart border. Flow voids traverse the tissue that is separated from the right atrium. Morphological and signal characteristics are that of thymus, thus excluding a mediastinal lymph node.

Like stated earlier on, PET-CT plays a crucial role in staging and treatment monitoring in lymphoma. Although HD and DLBC NHL are highly FDG avid and almost pose no problem in the use of [F18] FDG PET-CT for staging and evaluation of treatment response, other histological subtypes of NHL that have less FDG uptake must be known. They usually include primary cutaneous anaplastic large T-cell lymphoma, extranodal marginal zone lymphoma, and small lymphocytic lymphoma (3, 15). In such conditions, the interpreting physician should alert the referring physician that the evaluated subtype is less or not FDG avid, thus a follow-up study to assess response to therapy may be misleading and most likely not useful (11). Main pitfalls in lymphoma are due to thymic, bone marrow, splenic, and reactive uptake (1, 11). It is crucial to recognize thymic hyperplasia or physiologic activity in a young patient with FDG uptake in the anterior mediastinum (Figure 6). Although a normal variant in a young individual, the phenomenon is common and expected as a reaction to chemotherapy. Patients with myeloid hyperplasia following granulocyte colony-stimulating factor (G-CSF) therapy have diffuse bone marrow uptake. Similarly, during or immediately after chemotherapy, bone marrow activation also shows diffuse FDG uptake (Figure 7). While these instances may obscure possible bone marrow infiltration, diffuse uptake should not be interpreted as due to bone marrow involvement. When a focal bone marrow uptake is seen in the pelvis, particularly in the posterior ilium, one must first consider the history of BMB as the cause of single and focal uptake. Like bone marrow, the spleen will show diffuse increased uptake in post G-CSF or patients with concurrent infectious conditions. Lymphoma is primarily a nodal disease, and studies performed to evaluate response should be assessed with caution for residual and/or progression of disease. Mimic of lymph nodes may erroneously impact management, as the presence or absence of active nodes may alter treatment decision (Figure 8).

**Figure 9:**
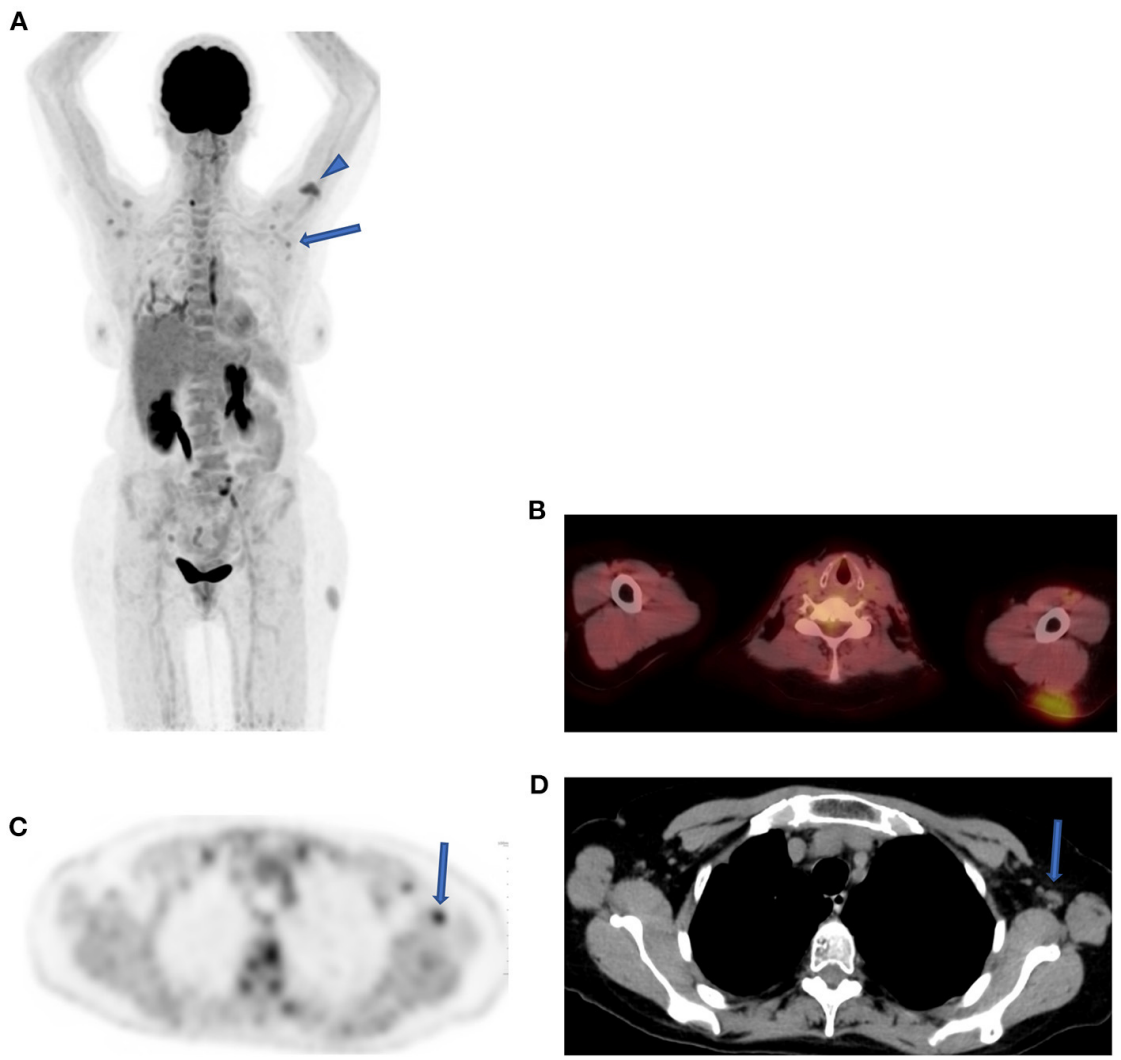
Reactive lymph nodes following SARS-CoV-2 (COVID19) vaccine. FDG PET MIPimage **(A)** in a patient demonstrating uptake in a cluster of lymph nodes in both axilla (solid arrow) and at the site of vaccine in left shoulder (head arrow) a month after the injection. Axial fused PET-CT shows the injection site in the subcutaneous area of the left shoulder **(B)**. Axial FDG PET shows the uptake in the largest lymph node with fatty center in the left axilla (solid arrow) **(C)**, and CT confirmed the characteristics of this reactive node **(D)**.

**Figure 10:**
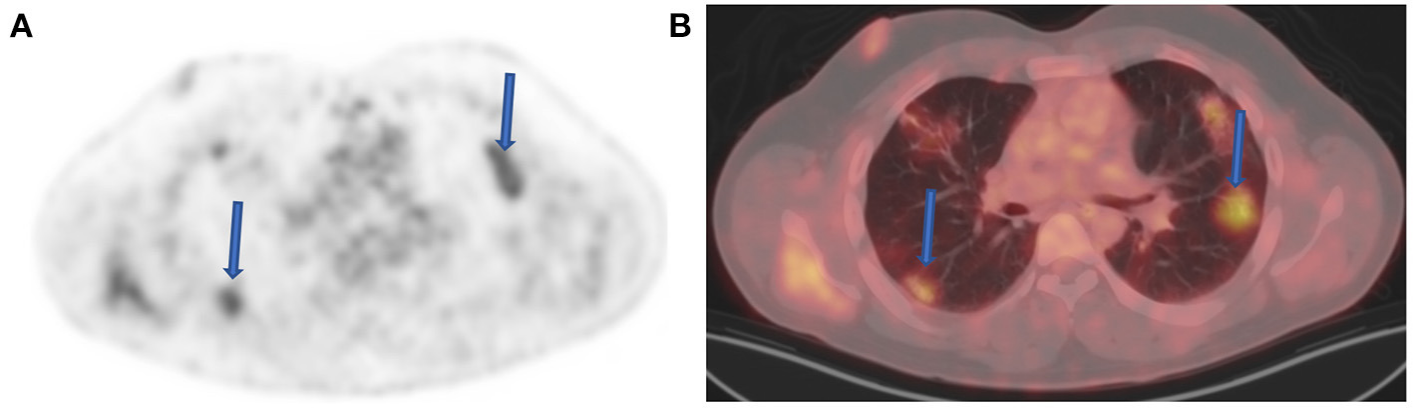
Ground-glass opacities (GGOs) with inflammation. FDG PET **(A)** and fused FDG PET-CT **(B)** images demonstrate increased FDG uptake within several bilateral foci of GGOs (arrows) in a 42-year-old male patient referred for post therapy evaluation. He indicated to have been diagnosed with coronavirus disease 2019 (COVID-19) infection 2 months before the study. Despite changes seen above, the patient was asymptomatic and did not have a lingering active chest infection.

Today, with the advent of SARS-CoV-2 (COVID19) infections, we are seeing an emergence of new entities that may mimic nodal pathology in the axilla and pulmonary infiltration. Patients with recent COVID-19 vaccine show active axillary lymph nodes on the side of the shoulder that received the vaccine (Figure 9). Immediately post COVID-19 infection, ground-glass opacification may be active with FDG uptake in the lungs (Figure 10).

Interpreting physicians must be aware of all these potential changes and their impact on PET-CT reports in patients with HD and NHL. Furthermore, the introduction of digital PET machines has resulted in increased sensitivity with a relative increased FDG uptake as compared to analog systems. In a recent prospective single-center study, Constantino et al. (16) showed higher lesion SUVs with digital systems in a head-to-head comparison with analog systems. However, when they applied specific protocols' reconstructions, the relative difference was minimized between analog and digital PET system (16).

## Conclusion

As PET-CT takes the center of imaging patients with lymphoma and the number of referrals is continuously increasing, it becomes imperative to recognize its limitations to improve patients' management. In this paper, we highlighted the normal variants, artifacts, and pitfalls related to lymphoma that should improve confidence of reporting physicians while reducing potential reporting misinterpretations.

## Author Contributions

MV: conception, write up, images collection, and revision. JM: write up and images collection. All authors contributed to the article and approved the submitted version.

## Conflict of Interest

The authors declare that the research was conducted in the absence of any commercial or financial relationships that could be construed as a potential conflict of interest.

## Publisher's Note

All claims expressed in this article are solely those of the authors and do not necessarily represent those of their affiliated organizations, or those of the publisher, the editors and the reviewers. Any product that may be evaluated in this article, or claim that may be made by its manufacturer, is not guaranteed or endorsed by the publisher.
